# Isolation and characterization of yeast for the production of rice wine with low fusel alcohol content

**DOI:** 10.1371/journal.pone.0260024

**Published:** 2021-11-17

**Authors:** Huawei Yuan, Wenhao Chen, Yuanlin Chen, Lian Wang, Chao Zhang, Wuyuan Deng, Liqiang Zhang, Guangqian Liu, Caihong Shen, Kai Lou, Songtao Wang

**Affiliations:** 1 Faculty of Quality Management and Inspection & Quarantine, Yibin University, Yibin, Sichuan, China; 2 Solid-state Fermentation Resource Utilization Key Laboratory of Sichuan Province, Yibin, Sichuan, China; 3 Faculty of Agriculture & Forestry and Food Engineering, Yibin University, Yibin, Sichuan, China; 4 Luzhou Laojiao Co., Ltd. Luzhou, Luzhou, Sichuan, China; 5 National Engineering Technology Research Center of Solid-state Brewing, Luzhou, Sichuan, China; University of Patras, GREECE

## Abstract

Fusel alcohols (FAs) are a type of flavor compound found in rice wine. An overly high FA content not only leads to spicy, bitter, and astringent taste but also has side effects. Therefore, screening for yeast that produce low FA contents has attracted much attention. Thirty-two yeast strains were isolated from fermenting material during *Luzhou*-flavor liquor production in this study. Strain YB-12 was selected as a suitable candidate for rice wine production. The strain was identified as a member of the genus *Meyerozyma* based on phylogenetic analysis using 26S rDNA gene sequences. The ability of strain YB-12 to produce ethanol was similar to that of *Saccharomyces cerevisiae* NRRL Y-567, while isobutanol and isoamyl alcohol production was only 53.96% and 50.23%, respectively, of that of NRRL Y-567. The FA yield of rice wine produced with strain YB-12 was reduced to 51.85% in a 20 L fermenter. These results demonstrate that strain YB-12 presents promising characteristics for use in the production of rice wine with a potentially low content of FAs.

## 1. Introduction

Rice wine is a generic name referring to alcoholic beverages made from rice in East Asia. The beverages are known as rice wine in the West because their alcohol content approximates that of a wine [1]. Rice wine is called sake in Japan, cheongju in Korea, and shaosingjiu in China. Sake is the national and traditional drink of Japan and is one of the most popular traditional fermented alcoholic drinks in the world [2]. It is prepared from rice using *koji*, and unique strains of *Saccharomyces cerevisiae* are used for these fermentations, generating products with ethanol contents (12–20%) and attractive flavors, aromas, and odors [3].

Making rice wines in Asia is more akin to brewing beer than to winemaking. Chinese rice wine and Japanese saké are produced by a method developed several thousand years ago in China. This system uses a cooked grain mass with a fungal culture termed *qu* or *jiuqu* in Chinese and *koji* in Japanese. Although recipes vary between countries, essentially, steamed rice porridge is treated with a starter of microorganisms grown on malted wheat cake (*nuruk*, Korea), rice cake (*koji*, Japan), red rice and/or wheat cake (*qu*, China), or cassava/rice cake (Vietnam) [4]. The liquefaction/saccharification of starch and fermentation of sugars occur simultaneously due to the presence of *Aspergillus, Rhizopus* and other amylolytic fungal species and *Saccharomyces cerevisiae* and other yeasts, respectively [5]. Rice wine produced by an amylolytic starter (*koji*) is not distilled, but the extract of fermented mash is filtered into clarified high-alcohol-content liquor [5].

Fusel alcohols (FAs) (also termed higher alcohols or fusel oils), which are alcohols with three or more carbon atoms, are byproducts of ethanol fermentation by *Saccharomyces cerevisiae* [6,7]. As the main flavor compounds, FAs are essential factors affecting the quality of rice wine. Isoamyl alcohol is one of the most abundant FAs in rice wine, followed by isobutanol, n-propanol, n-butanol, and β-benzoethanol, which together account for more than 99% of the total FA content [7]. The content and composition of FAs can make rice wine full-bodied, round, soft, and harmonious [8,9], which are organoleptic properties used to identify different wine products [6]. However, excessive FA contents can degrade the quality of rice wine and cause headaches, nervous hyperemia, and dizziness in consumers, in addition to giving the products a bitter-astringent taste or turbid/cloudy appearance [7,10,11]. Therefore, the FA content in rice wine must be controlled within a certain range, and the problem of high FA contents in the traditional brewing of rice wine has attracted wide attention.

As byproducts of yeast fermentation during rice wine brewing, FAs are produced by two pathways, namely, the amino acid synthesis pathway and the Ehrlich pathway, the latter of which plays a key role during nitrogen-limited growth of *S*. *cerevisiae* [6,12]. The FA content in fermentation products depends on the amino acid content in the medium and the uptake of amino acids by the yeast [13,14]. In this case, different strategies can be considered to reduce the FA content, including the modification of the medium composition, the selection of yeast strains with low FA production, the alteration of fermentation conditions, and even the application of procedures to remove FAs post fermentation [15].

In fact, various methods, such as low-temperature fermentation technology [16], nitrogen compensation during fermentation [17], and FA removal by nanofiltration after distillation [15], have been employed in rice wine production or the laboratory to reduce the FA content and control it to within a suitable range in the final products. However, these strategies only partially solve the problem and increase production costs. The most fundamental method is to use yeast strains with low FA production for fermentation. To date, *S*. *cerevisiae* strains with low FA production have been obtained by mutation breeding [18–20], ion implantation [21] and isolation-screening methods [22–24]. In these strains, the Ehrlich metabolic pathway was blocked. However, the breeding of yeast with low and stable FA production and high ethanol production is necessary to maintain the quality of rice wine since spontaneous mutagenesis in *S*. *cerevisiae* can change its production properties [25]. Currently, yeast isolated from fermented grains and other materials can be screened for low FA production using lactic acid medium because the production of FAs is inversely related to the metabolism of lactic acid [26].

Luzhou-flavor liquor, which is known commercially as Luzhou Laojiao (a famous Chinese trademark), refers to a kind of spirit distilled from fermented sorghum with a strong aroma and flavor, and this spirit is produced by Luzhou Laojiao Company, Ltd., in Luzhou city, Sichuan Province. Since the fermenting grains of Luzhou-flavor liquor are rich in microorganisms with industrial value, they have been previously screened for specific functional microorganisms with related and research applications [27]. In the present study, we screened for yeast with low FA production in fermented grains of Luzhou-flavor liquor for use in producing brewed rice wine. First, yeast strains with low FA and high ethanol production were selected. Then, the fermentation performance of the selected strains was further evaluated. The results demonstrated that the selected strains are potential candidates for use in traditional rice wine production technologies to reduce the side effects of FAs on the brewing of rice wine and improve brewing quality. This study is of significance for improving the quality of rice wine.

## 2. Materials and methods

### 2.1 Media used in the study

Four culture media were employed for yeast isolation in the present study: 1) proliferation medium (peptone, 5 g; yeast extract, 5 g; glucose, 20 g; soluble starch, 1 g; MgSO_4_·7H_2_O, 0.01 g; ZnSO_4_·7H_2_O, 0.4 g; K_2_HPO_4_, 2 g; CuSO_4_·5H_2_O, 0.05 g; distilled water, 1 L) for yeast propagation; 2) yeast peptone dextrose (YPD) medium (yeast extract, 10 g; peptone, 20 g; glucose, 20 g; agar, 15 g; dH_2_O, 1 L) for yeast isolation; 3) lactic acid (LA) medium (lactic acid, 40 g; (NH_4_)_2_SO_4_, 5 g; KH_2_PO_4_, 1 g; MgSO_4_, 0.5 g; yeast extract, 0.2 g; NaCl, 0.1 g; CaCl_2_, 0.1 g; agar, 15 g; dH_2_O, 1 L; pH 6.0); and 4) triphenyl tetrazolium chloride (TTC) medium (TTC, 0.1 g; lactic acid, 5 g; agar, 15 g; dH_2_O, 1 L; pH 6) for screening yeasts with low FA production. All media were sterilized at 121°C for 15 min.

*Koji* extract was used to test FA and ethanol production. The rice *koji* culture was prepared in an incubator (model HMJ-Ⅱ-300, Shanghai Yuejin Medical Instrument Co., Ltd., Shanghai, China) at 38°C and 95% humidity for 48 h [28]. *Koji* extract was prepared by immersing *koji* (50 g) in sterile water (150 mL) at 60°C for 18 h. The mixture was successively filtered through absorbent cotton and filter paper. Then, the obtained filtrate was adjusted to 10° Brix with sterile water before use.

### 2.2 Isolation and preliminary screening of the yeast

For yeast isolation, fermenting grains from a local Luzhou-flavor liquor distillery (Luzhou, China) were used. Fermenting grain samples (5 g) were inoculated into 100 mL of enrichment medium supplemented with antibiotics (ampicillin, tetracycline, chloramphenicol and erythromycin at final concentrations of 20 mg/L each) to suppress bacterial contaminants and cultured statically at 30°C for 48 h. Then, serial dilutions up to 10^−10^ were individually prepared for the cultures, and all of the dilutions were spread onto YPD agar plates (0.1 mL/plate). The inoculated plates were incubated at 30°C for 48~72 h. Single colonies with typical yeast characteristics were selected.

In the preliminary screening, the isolates were inoculated onto LA medium and incubated at 30°C for 3~5 d in the dark. The colony characteristics of the yeast were observed, and large single colonies with thick layers were selected because larger yeast colonies on this medium have lower FA production capacities [26]. Then, the selected single colonies were transferred to TTC medium, and the plates were further incubated in the dark at 30°C for 2~3 h. Different isolates showed different shades of red on this medium. Isolates with deep-red colonies were selected as the primary screened isolates because darker red yeast colonies on this medium have stronger alcohol production capacities [26,29]. The selected isolates were dispensed into fresh medium containing 20% (v/v) glycerol as a cryoprotectant and maintained at -80°C for further assays.

### 2.3 Fermentation experiments and secondary yeast screening

Thirty-two strains isolated from the fermenting grains of Luzhou-flavor liquor were assayed for their ability to produce FAs and ethanol. First, the isolates were activated. Isolates were grown statically in 50 mL of YPD medium in a 200 mL Erlenmeyer flask at 30°C for 48 h. Then, the inocula were prepared. The cultures mentioned above were incubated in thermostatic baths (model ZWY-211C, Shanghai Zhicheng Analytical Instrument Factory, Shanghai, China) at 30°C with shaking at 120 rpm for 24 h to obtain a culture containing no fewer than 3×10^8^ cells/mL, as determined by direct counting in a hemocytometer chamber. Subsequently, the yeast ability to produce FAs and ethanol was assayed. In total, of 1 mL of the inocula and 50 mL of *koji* extract were added to a 200 mL flask, and fermentations were performed statically for 120 h at 30°C. Samples (10 mL each) were collected after fermentation in all cases. Each sample was centrifuged at 12,000 rpm and 4°C for 5 min (MX-301, Tomy Industry Co. Ltd, Tokyo, Japan). After filtering the supernatant through a 0.2 μm membrane filter (SYT0116, Jinteng, China), the concentrations of FAs and ethanol were measured using gas chromatography (model 353B, GL Sciences, Tokyo, Japan), as previously described for FAs [30] and ethanol [31].

This assay was performed in triplicate, and the *S*. *cerevisiae* NRRL Y-567 strain was included as a reference. The yeast strains with lower FA (isoamyl alcohol and isobutyl alcohol) and higher ethanol production than the reference *S*. *cerevisiae* strain were selected for further analysis.

### 2.4 Detailed analysis of the flavor compounds generated by selected isolates

**Fig 1:**
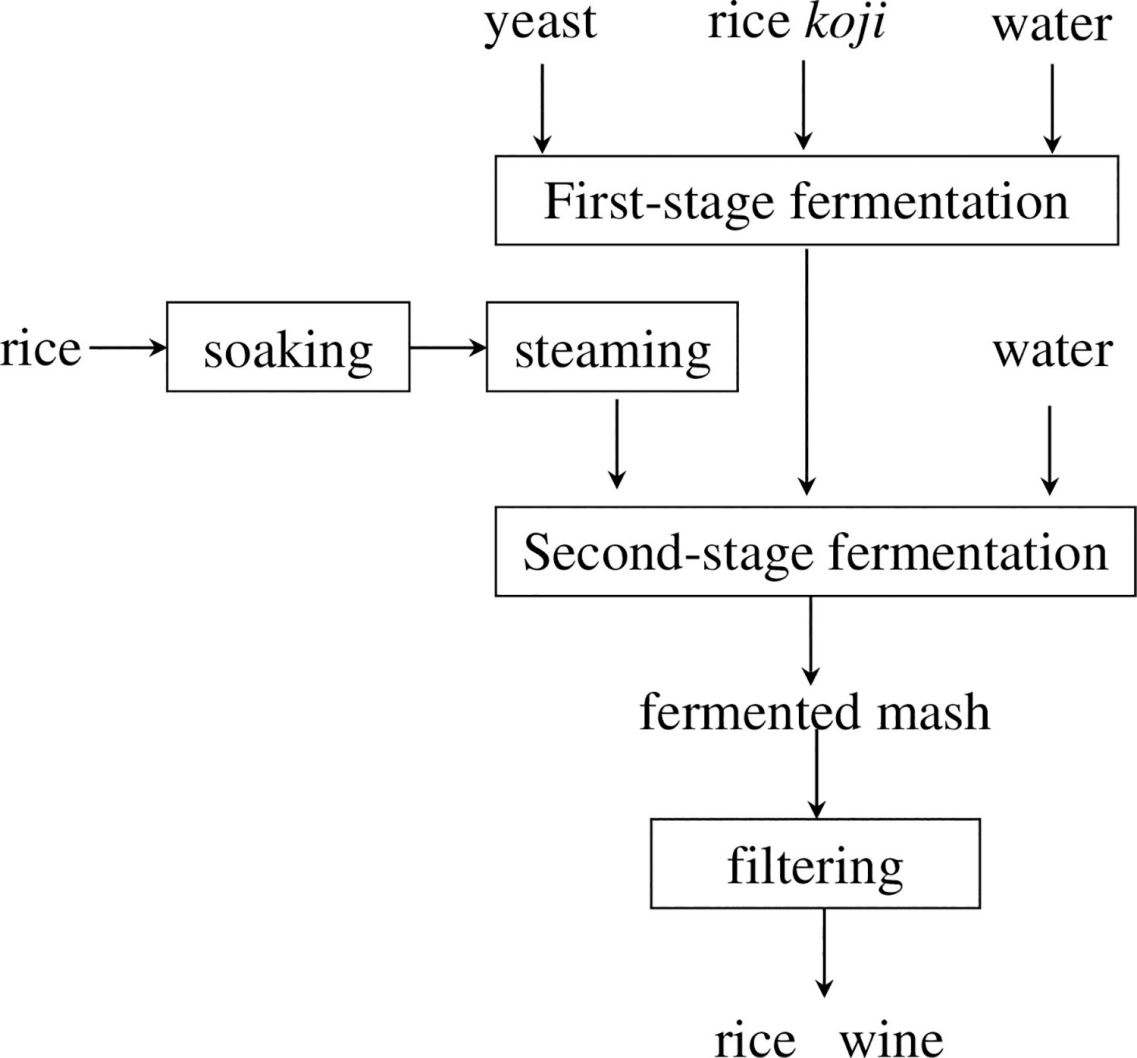
Flow diagram of rice wine production.

According to the screening results, five isolates, namely, YB-8, YB-12, YB-20, YB-27, and YB-31, and the reference strain *S*. *cerevisiae* NRRL Y-567 were selected for a comparative analysis of flavor compounds in rice wine. Rice wines were generated by fermentation, as shown in Fig 1. First-stage fermentation was carried out according to the following method: 82.5 g rice *koji*, 100 mL sterile water, and 1 mL of yeast inoculum (the concentration of yeast cells was not less than 3 × 10^8^ cells/mL) were added to a 1000 mL Erlenmeyer flask and mixed. The first stage was performed at 25°C for 3 d. Second-stage fermentation was initiated by adding steamed rice (rice dry weight, 250 g) and 450 mL of sterile water to the flask. The second stage was conducted at 25°C for 13 d with shaking once per day by hand. Finally, the fermented mash was filtered to obtain rice wine [32].

The compounds in the rice wines, including acetaldehyde, n-propyl alcohol, ethyl acetate, isobutyl alcohol, acetal, isoamyl acetate, n-butyl alcohol, isoamyl alcohol, ethyl lactate, ethyl caproate, acetic acid, β-phenethyl acetate, β-phenethyl alcohol and ethanol, were assayed using gas chromatography (model 353B, GL Sciences, Tokyo, Japan), as previously described [30].

### 2.5 Sensory evaluation of the rice wine

Sensory evaluation of rice wine was conducted according to the method described by Chen *et al* [33], Liu *et al* [34] and Xiang *et al* [35]. A panel of ten judges (five males and five females between 20 and 50 years old) were previously selected and trained according to ISO 8586–2 [36]. The 4 descriptors of rice wine were determined as color, flavor, taste and appearance to characterize the sensory properties of the rice wine fermented by the five isolates, YB-8, YB-12, YB-20, YB-27, and YB-31, and the reference strain *S*. *cerevisiae* NRRL Y-567. The total score of sensory evaluation was 10 points, of which, the scores for taste accounted for 6 points, flavor accounted for 2 points, and color and appearance accounted for 1 point each (Table 1). If the total score was 10, it indicated excellent quality [37]. In the first step, the panelists observed, tasted and sniffed the rice wine to recognize and record all the sensory attributes. In the second step, the panelists established final descriptors based on the provided standards. In the final step, the panelists were asked to express their judgments by quantifying each sensory score. Sensory analysis was conducted in a sensorial analysis room at 20°C. The 30mL of rice wine samples were placed in the same clear, tulip-shaped glass cup for evaluation. Water was provided for mouth rinsing between the evaluations of different samples to avoid a carryover effect of the aftertaste. The evaluation of these samples was carried out in randomized, swirl the contents well, and sniff the headspace vapor. Every sample was repeated three times.

**Table 1:** Sensory evaluation indexes of rice wine.

Item	Standard for evaluation	Score
Color	Shiny, light yellow.	1
Flavor	Fragrant with pleasant mellow and rich notes.	2
Taste	Soft, refreshing and pleasant taste, with moderate coordination of sour and sweet tastes.	6
Appearance	Clear, uniform.	1

### 2.6 Characterization and molecular taxonomy

The selected isolate YB-12 was cultured in YPD fermentation medium at 30°C for 24 h. A total of 10 mL of the fermented liquid was transferred to centrifuge tubes, and the cells were recovered by centrifugation at 10,000×g for 2 min. Genomic DNA was extracted from the recovered cells using the Yeast DNA Purification Kit (MasterPure™, Wisconsin, USA). The D1/D2 domain of the 26S rDNA region was amplified using the primers NL1 (5’-GCATATCAATAAGCGGAGGAAAAG-3’) and NL4 (5’-GGTCCGTGTTTCAAGACGG-3’). Amplification was conducted by PCR under the following conditions: initial denaturation at 95°C for 5 min, followed by 30 cycles of 94°C for 45 s, 55°C for 45 s, and 72°C for 1 min and a final extension at 72°C for 10 min. The amplified 26S rDNA D1/D2 region was sequenced using the Illumina NextSeq500 platform. The sequence acquired for the selected isolate was searched by BLAST against the GenBank nucleic acid sequence database to determine the similarity between the isolate sequence and the corresponding sequences of defined yeast species. Then, the phylogeny of the acquired sequence was analyzed by MEGA X. A phylogenetic tree was reconstructed by the neighbor-joining (NJ) method with distances computed using the maximum composite likelihood evolutionary model and bootstrapped with 1000 replicates [38].

### 2.7 Growth trials of the selected strain

The influences of the temperature, pH, glucose concentration and ethanol concentration on the selected strain and reference strain (*S*. *cerevisiae* NRRL Y-567) were investigated by growth trials. The tested variables were the temperature (20, 25, 30, 35, 40, 45, and 50°C), initial pH (5, 5.5, 6, 6.5, 7, 7.5, and 8), initial glucose concentration (10%, 15%, 20%, 25%, 30%, 35%, and 40% (w/v)), and initial ethanol concentration (4%, 6%, 8%, 10%, 12%, 14%, and 16% (v/v)). Cell growth experiments were performed in triplicate using the selected strain and reference strain (*S*. *cerevisiae* NRRL Y-567) for 24 h in a 200 mL flask containing 50 mL of YPD medium. Yeast growth in liquid culture was evaluated by turbidimetry according to the optical density at 600 nm measured in a UV-visible spectrophotometer (model UV8100B, Beijing Labtech Instrument Co., Ltd., Beijing, China).

### 2.8 Rice wine fermentation at different temperatures

First-stage fermentation was carried out using the selected strain and reference strain (*S*. *cerevisiae* NRRL Y-567). Then, second-stage fermentation was carried out at 20, 25, 30, 35, and 40°C for 13 d, as shown in Fig 1. FAs in the rice wine were assayed as described above.

### 2.9 Use of the selected yeast in a 20 L bioreactor

Inocula of the yeast isolate YB-12 obtained in this study and the reference strain *S*. *cerevisiae* NRRL Y-567 were prepared as described in 2.3 section. First-stage fermentation was performed statically at 25°C for 3 d by adding 20 mL yeast inoculum, 1650 g rice *koji* and 2000 mL sterile water to a 20 L bioreactor (Nagata Brewing Machinery Co., Ltd, Japan). Second-stage fermentation was initiated by adding steamed rice (rice dry weight, 5000 g) and 9000 mL of water to the bioreactor, followed by fermentation at 25°C for 13 d with daily shaking. Samples of 10 mL were collected every 2 d, and the concentrations of isoamyl alcohol and isobutyl alcohol were assayed. The flavor components of the rice wine were assayed as described above.

### 2.10 Statistical analysis

The mean values ± standard deviations of ethanol and FA concentrations were calculated from the triplicate samples. The data were analyzed using Duncan’s multiple range test with SPSS software (IBM Corp., Armonk, NY) to compare the different treatments.

## 3. Results and discussion

### 3.1 Isolation and screening of the yeast

In total, 124 yeast isolates were obtained from the fermented grains of Luzhou-flavor liquor on YPD agar plates during the preliminary screening. These isolates exhibited smooth, wet, and sticky surfaces and a soft texture, were easy to pick up, were mostly milky white or cream in color, had wine-like aromas, and exhibited other typical yeast-like characteristics. Strains with larger colonies on LA medium plates had higher activities of lactic acid dehydrogenase, which is the key enzyme in higher alcohol metabolism. Consequently, 32 strains with strong growth on LA medium and low FA production were selected from the secondary screen.

The appearance of the isolated colonies and their final ethanol, isoamyl alcohol and isobutyl alcohol concentrations produced using *koji* extract are shown in Table 2. The isolates with higher ethanol production were YB-20, YB-23, YB-31, YB-8, YB-12, YB-27, and YB-5, while the isolates with relatively low isobutanol and isoamyl alcohol production were YB-12, YB-27, YB-20, YB-31, and YB-5. Among these isolates, YB-8 produced not only relatively high concentrations of ethanol but also high concentrations of isoamyl alcohol and isobutyl alcohol. YB-25, YB-26 and YB-9 produced low concentrations of not only isoamyl alcohol and isobutyl alcohol but also ethanol. Only isolates YB-5, YB-12, YB-20, YB-27 and YB-31 produced low amounts of isobutyl alcohol and isoamyl alcohol while generating high concentrations of ethanol. Therefore, these five isolates were rescreened with the comprehensive consideration of rice wine fermentation. In studying the effects of different strains of *Saccharomyces cerevisiae* (N85 and XZ11) on the microbial composition in the process of rice wine fermentation, Zheng HL *et al*. [39] found that different *Saccharomyces cerevisiae* strains could influence microbial compositions, especially affecting the growth of *Lactobacillus brevis* and *Pantoea ananatis*. These changes in the microbial community structure contributed to remarkable differences in the contents of lactic acid, esters, alcohols, and aldehydes [39]. This study expands the idea of improving the quality of rice wine by controlling the microbiome. It is important that more yeasts are selected and tested for their influence on the flavor compounds of rice wine.

**Table 2:** Screening results for yeast isolates.

Isolate code	Appearance of the colony	Concentration of alcohols in the culture		
		Ethanol (%, v/v)	Isoamyl alcohol (mg/L)	Isobutyl alcohol (mg/L)
YB-1	Round, yellowish	4.24±0.15 k	150.43±2.48 jkl	67.92±1.02 mno
YB-2	Round, buttery yellow	3.46±0.06 f	164.16±3.52 p	71.10±0.75 pqr
YB-3	Round, yellowish	3.52±0.07 fg	170.64±5.24 q	72.68±0.42 r
YB-4	Round, yellowish	2.64±0.11 d	154.63±3.07 lmn	69.40±0.57 nopq
YB-5	Round, creamy	4.82±0.12 n	137.46±3.76 hi	47.27±2.14 c
YB-6	Round, buttery yellow	3.27±0.10 e	132.45±3.46 gh	65.71±1.37 klm
YB-7	Round, buttery yellow	3.85±0.14 hi	160.84±1.40 op	68.18±2.73 mno
YB-8	Round, yellowish	5.17±0.03 p	174.27±1.05 qr	51.04±2.01 d
YB-9	Round, buttery yellow	1.97±0.05 b	104.63±2.24 d	53.21±0.89 de
YB-10	Round, creamy	3.72±0.04 gh	145.76±3.67 j	68.62±1.26 nop
YB-11	Round, buttery yellow	2.81±0.09 d	156.45±4.12 mno	69.34±1.33 nopq
YB-12	Round, creamy	5.12±0.16 op	75.35±3.02 a	35.72±1.47 a
YB-13	Round, buttery yellow	4.50±0.13 lm	157.89±2.45 no	70.29±1.14 opqr
YB-14	Round, buttery yellow	1.53±0.08 a	148.94±2.94 jk	59.34±0.76 gh
YB-15	Round, buttery yellow	4.27±0.09 k	154.27±4.13 lmn	62.53±0.92 ij
YB-16	Round, yellowish	3.76±0.12 h	176.37±3.18 r	67.09±1.84 lmn
YB-17	Round, buttery yellow	4.30±0.15 k	132.54±2.54 gh	71.35±1.27 qr
YB-18	Round, buttery yellow	3.25±0.07 e	151.28±3.41 klm	64.27±1.45 jk
YB-19	Round, creamy	3.54±0.12 fg	146.49±1.83 jk	71.61±1.27 qr
YB-20	Round, yellowish	5.25±0.14 p	104.23±4.20 d	58.64±1.43 g
YB-21	Round, creamy	2.43±0.05 c	127.48±1.57 g	61.57±1.39 hi
YB-22	Round, buttery yellow	4.02±0.16 ij	135.06±3.75 hi	75.63±1.40 s
YB-23	Round, creamy	5.21±0.07 p	151.23±2.71 klm	55.42±1.65 ef
YB-24	Round, creamy	3.68±0.13 gh	139.65±2.45 i	57.70±1.62 fg
YB-25	Round, yellowish	4.17±0.09 jk	96.34±1.12 c	47.87±0.78 c
YB-26	Round, yellowish	4.32±0.08 kl	87.26±1.76 b	51.39±1.09 d
YB-27	Round, creamy	4.94±0.21 no	110.24±2.79 e	39.75±1.34 b
YB-28	Round, creamy	4.52±0.18 m	128.25±1.43 g	65.22±1.38 kl
YB-29	Round, creamy	1.85±0.03 b	107.64±1.35 de	58.64±1.43 g
YB-30	Round, creamy	3.67±0.04 gh	138.47±1.27 i	63.56±1.21 ijk
YB-31	Round, creamy	5.18±0.15 p	120.36±1.89 f	67.80±1.30 mno
YB-32	Round, creamy	3.52±0.06 fg	112.47±2.78 e	71.29±2.14 qr

Note: the initial reducing sugar concentration in the rice *koji* juice medium was 95.62 g/L. Values followed by different *lowercase letters* within each column are significantly different at *P* = 0.05 according to Duncan’s multiple range test.

The FAs and other related flavor substances in the rice wine fermented by isolates YB-5, YB-12, YB-20, YB-27, and YB-31 and the reference strain are shown in Table 3. The yields of isobutanol and isoamyl alcohol from YB-12-mediated fermentation (72.70 mg/L and 162.48 mg/L, respectively) were significantly lower than those produced by other isolates and corresponded to 53.96% and 50.23%, respectively, of the concentrations produced by the reference strain of *S*. *cerevisiae*. Chen *et al*. [26] isolated yeast MT-14, which could produce 51.0% of the isobutyl alcohol, 53.4% of the isoamyl alcohol and nearly the same concentration of ethanol compared to the *Schizosaccharomyces pombe* strain ATCC 16979. Liang *et al*. [23] identified yeast strain JH301, in which FAs produced only 117.12 mg/L FAs and had an alcohol production of 18.5%.

**Table 3:** Fermentation scores, ethanol concentrations and amounts of flavor substances produced by selected yeasts in fermented rice wine (mg/L).

Flavor compound (mg/L)	Yeast					
	Reference strain	YB-5	YB-12	YB-20	YB-27	YB-31
Acetaldehyde	60.12±2.10 b	56.24±2.43 a	63.23±1.64 bc	65.82±2.30 cd	52.73±2.07 a	67.18±1.94 d
n-Propyl alcohol	90.25±4.13 c	87.40±2.07 c	62.47±3.25 a	62.46±3.12 a	97.38±3.20 d	70.36±2.56 b
Ethyl acetate	34.28±1.08 c	38.67±0.74 d	35.25±1.13 c	39.74±1.74 d	27.40±0.83 a	31.75±1.17 b
Isobutyl alcohol	134.72±2.73 e	128.59±3.16 d	73.56±2.38 a	107.27±2.93 c	125.56±1.62 d	96.08±2.37 b
Acetal	23.14±0.56 a	27.46±1.07 b	22.76±0.87 a	35.29±1.05 d	32.52±1.21 c	34.93±0.87 d
Isoamyl acetate	3.87±0.02 f	2.25±0.03 a	3.79±0.06 e	2.53±0.04 c	2.78±0.05 d	2.36±0.02 b
n-Butyl alcohol	11.96±0.04 d	14.84±0.01 f	8.25±0.15 a	9.57±0.05 c	14.66±0.01 e	9.37±0.03 b
Isoamyl alcohol	323.47±4.68 e	325.23±5.42 e	163.97±3.47 a	226.71±5.86 b	304.76±5.46 d	278.59±3.24 c
Ethyl lactate	ND	ND	ND	ND	ND	ND
Ethyl caproate	0.95±0.03 d	1.03±0.02 e	0.94±0.01 d	0.86±0.02 c	0.65±0.01 a	0.72±0.02 b
Acetic acid	1.04±0.04 b	0.85±0.01 a	0.85±0.02 a	1.13±0.03 c	1.26±0.02 d	1.03±0.02 b
β-Phenethyl acetate	24.35±0.78 b	27.28±0.62 c	26.32±0.48 c	20.71±0.64 a	23.85±0.67 b	21.50±0.65 a
β-Phenethyl alcohol	111.46±2.32 b	137.60±3.45 d	114.78±3.25 bc	115.06±1.62 bc	106.25±2.35 a	117.96±3.51 c
Ethanol (%v/v)	13.28±0.40 a	14.24±0.34 b	14.32±0.24 b	14.91±0.35 c	13.79±0.27 ab	15.14±0.29 c
Sensory score	9.03±0.03 e	8.76±0.07 d	9.13±0.05 f	8.25±0.02 c	7.84±0.06 b	7.67±0.05 a

Note: Values followed by different *lowercase letters* within a single column are significantly different at *P* = 0.05 according to Duncan’s multiple range test. ND: not detected.

To obtain a low-FA-yielding *Saccharomyces cerevisiae* strain, atmospheric and room-temperature plasma (ARTP) mutagenesis were used to mutate the yeast, and compared with the origin strain CF4, the FA production of the *Saccharomyces cerevisiae* mutant ARTP5 was reduced by 20% [40]. Gene knockout techniques are useful molecular tools for achieving decreased production of higher alcohols by *Saccharomyces cerevisiae* in rice wine fermentation. BAT1 and BAT2 single- and double-gene-deletion mutant strains were constructed from the industrial yeast strain RY1 to decrease higher alcohol production during Chinese rice wine fermentation. The results showed that the BAT2 single-gene-deletion mutant strain produced the best improvement in the production of FAs, while the remaining strains showed normal growth and fermentation characteristics [41]. Furthermore, a BAT2 single-gene-deletion diploid engineered strain RY1-Δbat2 was constructed and produced low levels of isobutanol and isoamylol, at 92.40 and 303.31 mg/L, respectively, in a simulated fermentation of Chinese rice wine, which were 33.00 and 14.20% lower than those produced by the parental strain RY1 [41]. The disadvantage of mutant and recombinant yeast strains is their instability, which hinders their application in fermented alcoholic beverages.

Isolate YB-12 presented similar levels of FAs, but it exhibited greater ethanol production and a higher sensory evaluation score than the reference strain. Although the ethanol yields from all isolates were greater than that of the reference strain and the production of FAs by other isolates was similar to that by YB-12, the other tested isolates received lower sensory scores than the reference strain, meaning that they decreased the rice wine quality. Therefore, only isolate YB-12 was selected for follow-up experiments.

### 3.2 Morphological observation and molecular identification of the isolate YB-12

**Fig 2:**
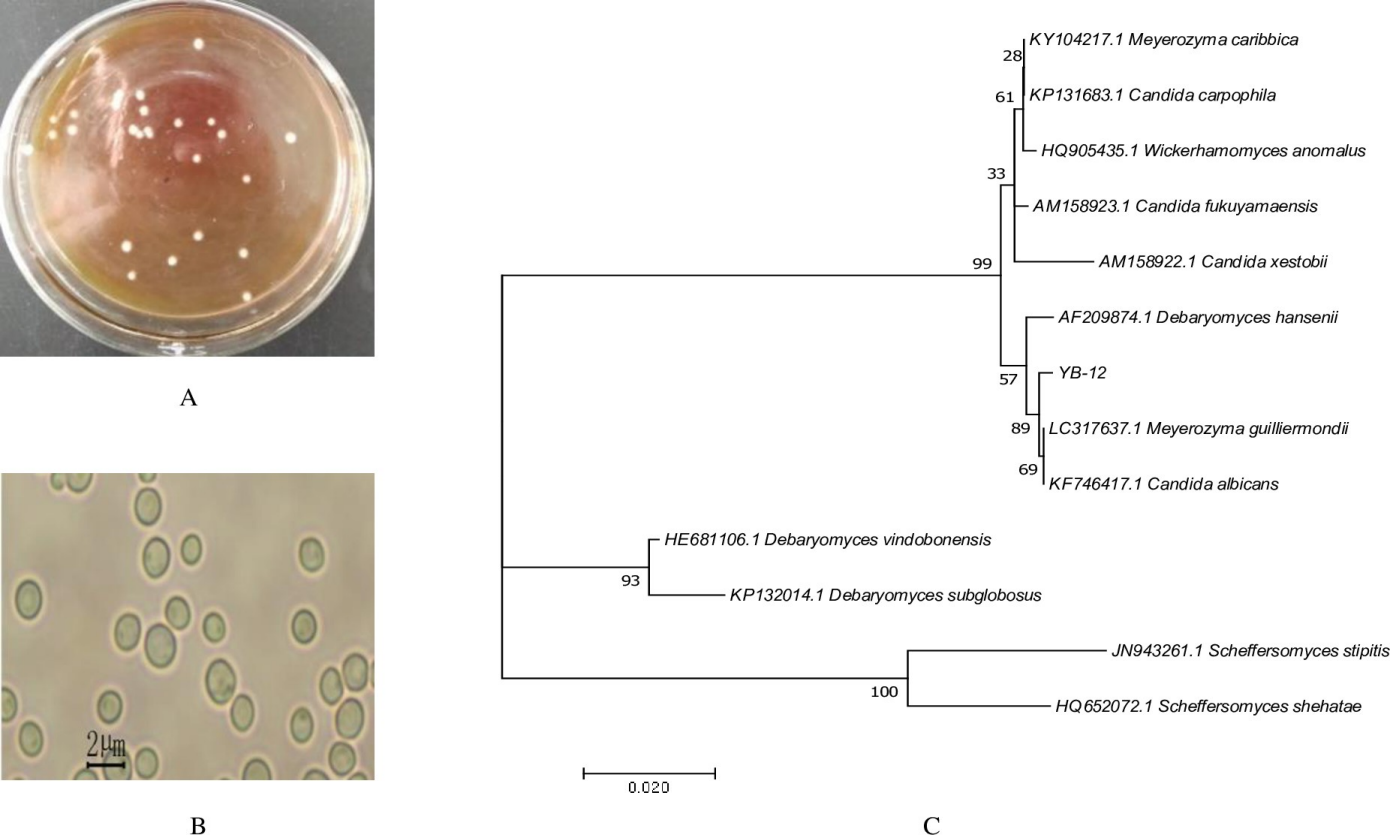
Morphological and molecular characterization of YB-12. A: Colony morphology. B: Cellular morphology. C: Phylogenetic trees of partial 26S rRNA gene sequences showing the relationships between YB-12 and its relatives. The tree was constructed via the NJ method. The scale bar represents 2% nucleotide substitutions. Bootstrap values are indicated at each node.

The colony and cell morphology and phylogenetic position of YB-12 in the NJ tree constructed with its 26S rDNA sequence are presented in Fig 2. The colonies of YB-12 on YPD agar plates were raised, sticky, white, and circular with smooth, wet surfaces and complete edges (Fig 2A). The cells of YB-12 were elliptical or oval under a high-magnification lens (40×) and exhibited even sizes (Fig 2B).

In the phylogenetic tree, YB-12 was identified by sequencing the 26S rDNA D1/D2 domain and comparing it with the sequences of type strains in the NCBI database (Fig 2C). The GenBank accession number for YB-12 is MW599291. The alignment results of the rDNA sequences of the isolate showed that the sequence of strain YB-12 has 99.83% similarity with *Meyerozyma gulliermondii* (LC317637.1). As shown in Fig 2, isolate YB-12 shares the same clade cluster with this species in the phylogenetic tree of D1/D2 26S rDNA sequences, further suggesting that it is a member of the genus *Meyerozyma*.

This identification and the screening results showed that YB-12 is a novel candidate for use in the production of rice wine with low FA production and preservation of the traditional flavor (Table 2). Additionally, our selected strain is different from that reported by Chen *et al*. [26], who identified a low-FA-yield yeast from *Daqu* and Z*aopei* of Maotai-flavor liquor, which was *Schizosaccharomyces pombe*, and that of Liang *et al*. [23], who isolated a low-FA- and low-urea-producing yeast from Hong Qu, which was *Saccharomyces cerevisiae*. The yeast strain JJND-072 with low fusel oil (0.81 g/L) and high alcohol production (29.3%) was screened out, and its culture formula and fermentation conditions were optimized [42]. Yeast YB-12 was isolated from the fermenting grains of Luzhou-flavor liquor. These discrepancies may have been caused by the differences in raw materials used for yeast screening.

### 3.3 Effect of fermentation conditions on the growth of YB-12 and the reference strain

**Fig 3:**
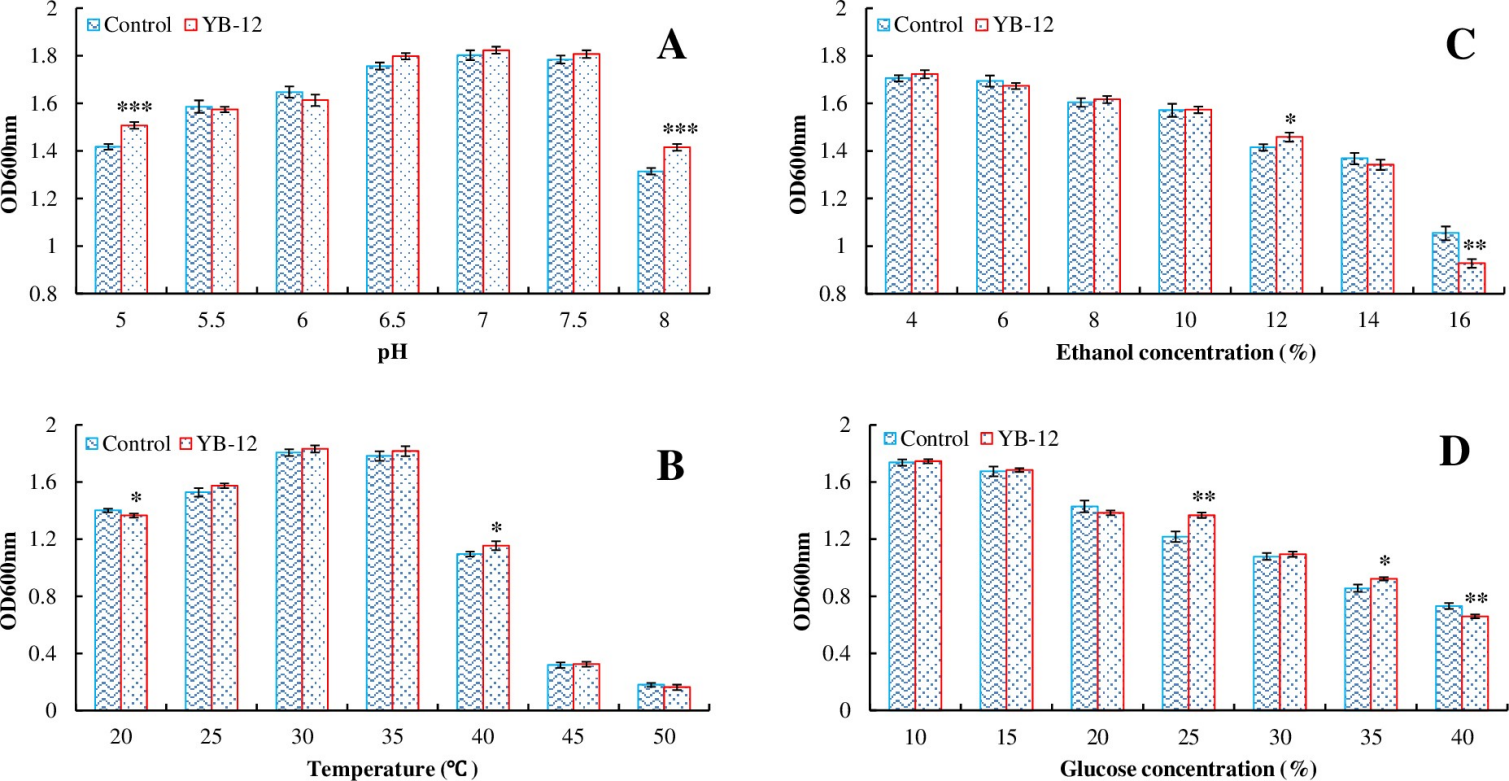
Effects of pH (A), temperature (B), ethanol concentrations (C) and glucose concentrations (D) on the growth of YB-12. Significance levels: *, *P* = 0.05; **, *P* = 0.01; ***, *P* = 0.001.

In general, similar growth tendencies were observed between the tested strain YB-12 and the reference strain *S*. *cerevisiae* NRRL Y-567 in response to culture conditions in YPD medium at 30°C. Both YB-12 and NRRL Y-567 presented maximum growth at an initial pH of 6.5~7.5, while the growth of YB-12 was significantly better than that of the reference strain at pH 5 and 8 (*P* = 0.001), although it reached only 60% of its maximum growth (Fig 3A).

Both YB-12 and NRRL Y-567 grew at a fairly broad temperature range (20~50°C), with an optimum temperature of 30~35°C, while the growth of both strains was decreased at lower or higher temperatures (*P* = 0.05). Compared to the reference strain, the growth of YB-12 at 20°C was lower and that at 40°C was better (*P* = 0.05) (Fig 3B). This result is different from that reported by Liang *et al*. [18], who identified a yeast with excellent growth at 10°C in a screen for fermentation at low temperatures, which had been bred to adapt to the local low-temperature environment for spontaneous fermentation.

The growth of yeast YB-12 and the reference strain gradually decreased with increasing concentrations (4~14%, v/v) of ethanol in the medium, and significant inhibition of both strains was observed at an initial ethanol concentration of 8% (v/v). The growth of YB-12 in 12% and 16% (v/v) ethanol was significantly better and worse, respectively, than that of the reference strain (*P* = 0.05, 0.01) (Fig 3C). In addition, the growth of YB-12 and the reference strain was not significantly affected by initial glucose concentrations of up to 15% (w/v) but was inhibited with a further increase in the glucose concentration. Moreover, both tested yeasts could still grow when the glucose concentration increased to 40% (w/v) (Fig 3D). The growth difference between the YB-12 and control strains (*S*. *cerevisiae*) was nonsignificant according to error analyses.

### 3.4 Effect of rice wine fermentation temperature on FA production

**Fig 4:**
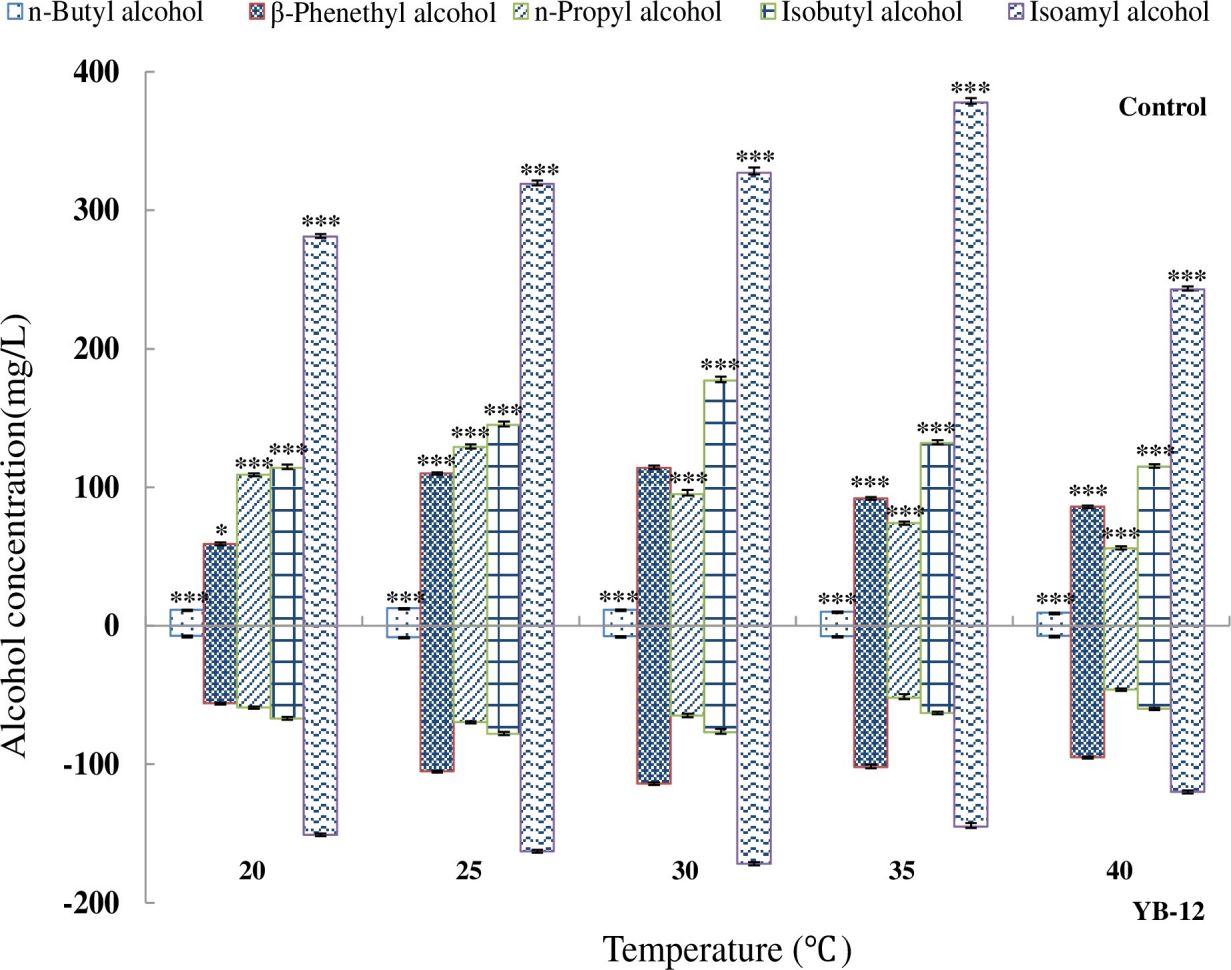
Effect of the fermentation temperature on FA production by the reference strain (A) and yeast YB-12 (B). Significance levels: *, *P* = 0.05; **, *P* = 0.01; ***, *P* = 0.001.

YB-12 produced the highest amount of FAs at 30°C, while the reference *S*. *cerevisiae* strain produced the highest amount of FAs at 35°C (Fig 4). At high fermentation temperatures, the activity of the yeast was high, and the fermentation speed was fast, but they were more susceptible to decay and contamination. On the other hand, the situations were reversed at low fermentation temperatures, and the yeast maintained their activity for a longer duration, which is better for producing rice wine with a good aroma. In the production process, a low fermentation temperature, normally 25°C, is considered optimal.

Similar results were observed previously by Yamamoto *et al*. [43], who used the yeast *Heisei miyazaki* MF062 for fermentation at different temperatures and obtained lower isoamyl alcohol and isobutyl alcohol levels at both 20°C and 38°C, with the highest levels at 28°C. The n-butyl alcohol content was the highest at 20°C and decreased at 28°C and 38°C. However, the β-phenethyl alcohol content was the lowest at 20°C and increased at 28°C and 38°C. The FA yields of YB-12 and the reference strain were significantly different (*P* = 0.001), except for β-phenethyl alcohol at 20°C (*P* = 0.05). At all tested temperatures, YB-12 produced half as much FA as the reference strain of *S*. *cerevisiae* (Fig 4).

### 3.5 Rice wine fermentation in a 20 L bioreactor

**Fig 5:**
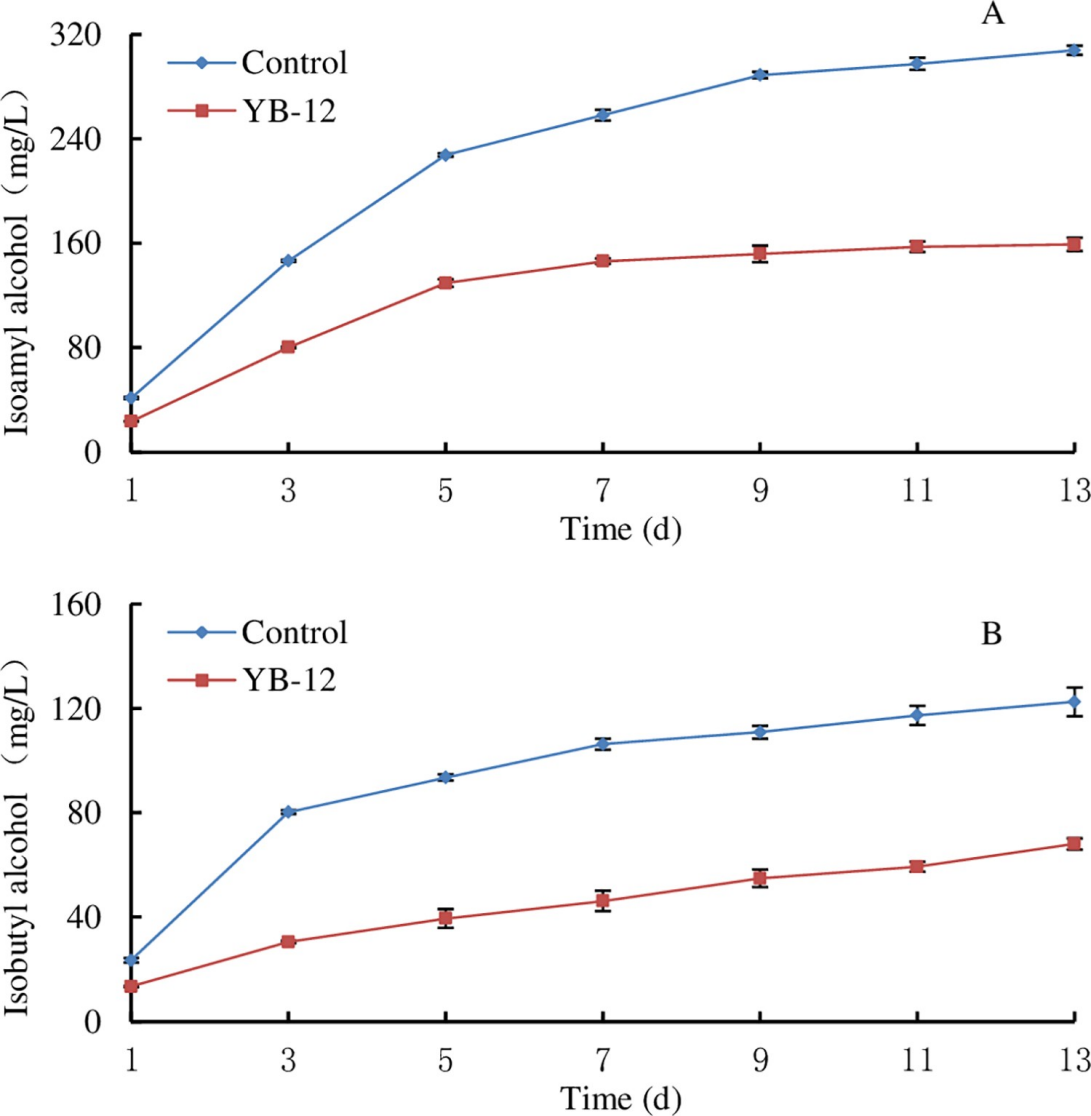
Changes in isoamyl alcohol (A) and isobutyl alcohol (B) during fermentation by YB-12 and the reference strain in a 20 L bioreactor.

In the 20 L fermenter, with fermentation at 25°C (Fig 5), the levels of isoamyl alcohol and isobutyl alcohol in the products of YB-12 and the *S*. *cerevisiae* reference strain significantly increased in the early stage of fermentation (1~5 d). However, the amounts of these two compounds in the YB-12 culture were only half of those in the culture of the reference strain. After 9 d of fermentation, a significant increase in isoamyl alcohol content was not observed, while the isobutyl alcohol content continued to increase slowly. These results are in agreement with the studies reported by Yuan *et al*. [44], who observed that isoamyl alcohol and isobutyl alcohol levels increased continuously in almost all fermentation processes. According to the influences of different raw materials, fermentation acidities, fermentation pressures, *koji* and nitrogen sources on FAs during fermentation, the complete fermentation process for low yields of fusel oil was optimized [42], which indicated that fermentation conditions were very important for low FA and high wine yields.

Microorganism species and inoculation fermentation methods have great influences on the physicochemical and flavor properties of rice wine. Chen LH *et al*. [45] investigated the microbial interactions and physicochemical and aroma changes of rice wine with different inoculation strategies using *Wickerhamomyces anomalus* and *Saccharomyces cerevisiae*. The sequential cofermentation consumed relatively more sugar and resulted in a higher ethanol content, causing reduced thiols and increased alcohols, esters, phenylethyls, and terpenes, which was more conducive to improving rice wine flavor than simultaneous cofermentation. Moreover, simultaneous cofermentation increased the fatty aroma of rice wine, while sequential cofermentation increased mellow and cereal-like flavors. These results confirmed sequential cofermentation with *S*. *cerevisiae* and *W*. *anomalus* as an option for the future production of rice wine with good flavor and quality.

The effects of the rice variety and fermentation time on rice wine quality were determined by Asgedom WH *et al*. [46]. Significant differences at P<0.05 were observed among rice varieties with respect to different wine quality parameters, and the maximum pH (4.98), total soluble solids (3.83°Brix) and overall sensory acceptance (4.32) were recorded for rice wine prepared from the X-Jigna variety after 5 days of fermentation. Wine prepared from the Gomera variety after 7 days of fermentation recorded the highest alcohol content (15.47%), followed by the X-Jigna variety after the same fermentation time (14.90%). In comparison, wine from the Gomera rice variety with 6 days of fermentation time was found to have the lowest overall acceptance (3.84). This is different from our results because the rice variety and yeast strain that we used were different.

The flavor components of the rice wine produced by YB-12 and the *S*. *cerevisiae* reference strain in the 20 L fermenter (Table 4) were the same. The produced amounts of acetaldehyde, ethyl acetate, isoamyl acetate, and β-phenylethyl acetate (*P* = 0.05) were similar between the two strains. However, the production of n-propyl, isobutyl, n-butyl, isoamyl and β-phenethyl alcohols was significantly lower (*P* = 0.05) in the YB-12 culture than in the culture of the reference strain. The important flavor components of rice wine, namely, β-phenethyl acetate, β-phenethyl alcohol and isoamyl acetate, presented concentrations of 22.01, 117.39 and 3.84 mg/L, respectively, in rice wine fermented by YB-12, which were consistent with the reported levels in rice wine (25.34, 133.96 and 4.00 mg/L, respectively) [40]. The production of isobutanol and isoamyl alcohol by YB-12 reached only 53.96% and 50.23%, respectively, of the levels produced by *S*. *cerevisiae*. The production of isobutanol and isoamyl alcohol was 89.28±1.85 mg/L and 192.03±4.19 mg/L, respectively, by YB-12 and 175.67±2.26 mg/L and 382.45 ±4.52 mg/L, respectively, by *S*. *cerevisiae*. In addition, the total production of isobutyl alcohol, isoamyl alcohol, n-propyl alcohol, and n-butyl alcohol by YB-12 was 51.85% that by *S*. *cerevisiae*.

**Table 4:** Flavor components of rice wine fermented in a 20 L fermenter (mg/L).

Flavor compound	Reference	YB-12
Acetaldehyde	24.86±0.78 a	25.63±0.92 a
Ethyl acetate	31.47±1.03 a	30.87±1.14 a
Acetal	3.98±0.094 a	4.15±0.078 a
n-Propyl alcohol	154.16±1.46 a	86.74±1.86 b
Isobutyl alcohol	175.67±2.26 a	89.28±1.85 b
Isoamyl acetate	3.78±0.046 a	3.84±0.072 a
n-Butyl alcohol	18.26±0.24 a	10.76±0.17 b
Isoamyl alcohol	382.45±4.52 a	192.03±4.19 b
β-Phenethyl acetate	21.97±0.031 a	22.01±0.033 a
β-Phenethyl alcohol	120.54±0.55 a	117.39±0. 46 b

Note: Values followed by different *lowercase letters* within a single column are significantly different at *P* = 0.05 according to Duncan’s multiple range test.

Zhong X *et al*. [47] used nitrogen compensation to reduce FAs, with Chinese rice wine as an experimental model. FAs, including isobutyl alcohols, isoamyl alcohols, and β-phenethyl alcohols, were significantly decreased by 19.27, 23.03 and 19.43%, respectively, when 200 mg/L (NH_4_)_2_HPO_4_, 5% (w/v) yeast, and 11% wheat *koji* were added to the fermentation broth. Meanwhile, important quality parameters remained unchanged, including free amino acids, organic acids, biogenic amines, and esters. These results suggest that ammonium compensation can effectively decrease FAs in Chinese rice wine. The molecular mechanism of ammonium compensation in reducing the content of FAs involved the reversal of the carbon flow that would have gone to the FA synthesis pathway to the TCA cycle, which thereby decreased the content of FAs [48]. The effects of nitrogen compensation on YB-12 in the process of rice wine fermentation should be determined in the future.

The determination of the ratios of the main FA components in rice wine produced by YB-12 and *S*. *cerevisiae* (Table 5) showed that there were no significant differences in the A/P, A/B, and P/B ratios between the products of YB-12 and those of *S*. *cerevisiae*. The A/P, A/B, and P/B ratios influence the flavor of rice wine, and the values were consistent with the reported ranges of 0.6~7.0, 0.8~2.7 and 0.3~4.0, respectively, in mature *moromi* fermented with rice *koji* [39]. In addition, the sensory evaluation scores indicated that the rice wine produced by yeast YB-12 was better than that produced by *S*. *cerevisiae*, demonstrating that the YB-12 strain reduced the FA content and had a positive effect on the flavor of rice wine. Lab-scale fermentation of yellow rice wine was conducted with the engineered haploid yeasts Na-Y and Na-I. The results showed that the concentration of isoamyl alcohol was almost invariable between the engineered strain Na-Y and the parental haploid. In contrast, the content of isoamyl alcohol was reduced by 16.16% with the engineered strain Na-I [49], which means that the isoamyl alcohol synthesis mechanism in *Saccharomyces cerevisiae* should be further investigated.

**Table 5:** Sensory evaluation and ratios of FA contents in rice wine fermented in a 20 L fermenter.

Sample	Score	A/P	A/B	P/B
Control (*S*. *cerevisiae* NRRL Y-567)	8.95±0.05	2.48	2.18	0.88
*Candida guilliermondi* YB-12	9.23±0.03	2.21	2.15	0.97

Note: A: isoamyl alcohol, B: isobutyl alcohol, P: n-propyl alcohol.

## 4. Conclusion

The fermented grains of Luzhou-flavored liquor were used to isolate 32 low-FA-producing yeast isolates, of which 5 had the ability to produce lower levels of FAs and higher levels of ethanol. One of these isolates, YB-12, had an ethanol production capacity that was the same as that of the reference strain *S*. *cerevisiae* NRRL Y-567, while the concentrations of isobutyl alcohol and isoamyl alcohol produced by YB-12 reached only 53.96% and 50.23%, respectively. We also observed that isolate YB-12 shared 99.83% similarity with strain *Meyerozyma guilliermondii* and was identified as a member of the genus *Meyerozyma*. Strain YB-12 was highly sensitive to changes in temperature, with an optimal temperature for growth of 30–35°C and an optimal initial pH of 6.5–7.5; Additionally, the best growth was achieved using 10% (v/v) ethanol and 20% initial sugar. The growth characteristics of YB-12 were similar to those of the reference strain. The total FA yield of strain YB-12 in a 20 L fermenter was 51.85% that of the reference strain; moreover, the sensory evaluation scores and ratios of FA contents in the rice wine were not significantly different between the two strains. The process was successfully scaled up from the laboratory scale to a 20 L bioreactor. Strain YB-12 was selected and evaluated for its potential to produce rice wine with a low FA content and suitable flavor. Hence, strain YB-12 presents promising characteristics for use in the production of rice wine with potentially low FA contents.

## Supporting information

S1 Table Screening results for yeast isolates. (XLSX)

S2 Table Effects of pH (A), temperature (B), ethanol concentrations (C) and glucose concentrations (D) on the growth of YB-12. Significance levels: *, *P* = 0.05; **, *P* = 0.01; ***, *P* = 0.001.
(XLSX)

S3 Table Fermentation scores, ethanol concentrations and amounts of flavor substances produced by selected yeasts in fermented rice wine (mg/L). (XLSX)

S4 Table Effect of the fermentation temperature on FA production by the reference strain (A) and yeast YB-12 (B). Significance levels: *, *P* = 0.05; **, *P* = 0.01; ***, *P* = 0.001.
(XLSX)

S5 Table Flavor components of rice wine fermented in a 20 L fermenter (mg/L). (XLSX)

S6 Table Changes in isoamyl alcohol (A) and isobutyl alcohol (B) during fermentation by YB-12 and the reference strain in a 20 L bioreactor.
(XLSX)

S7 Table Sensory evaluation and ratios of FA contents in rice wine fermented in a 20 L fermenter. (XLSX)
